# Cognitive and Affective Aspects of Creative Option Generation in Everyday Life Situations

**DOI:** 10.3389/fpsyg.2016.01132

**Published:** 2016-08-03

**Authors:** T. Sophie Schweizer, Katja M. Schmalenberger, Tory A. Eisenlohr-Moul, Andreas Mojzisch, Stefan Kaiser, Joachim Funke

**Affiliations:** ^1^Institute of Medical Psychology, Medical Faculty, University of HeidelbergHeidelberg, Germany; ^2^Department of Psychiatry, University of North Carolina at Chapel Hill, Chapel HillNC, USA; ^3^Institute of Psychology, Department of Educational and Social Sciences, University of HildesheimHildesheim, Germany; ^4^Department of Psychiatry, Psychotherapy and Psychosomatics, Psychiatric Hospital, University of ZurichZurich, Switzerland; ^5^Institute of Psychology, University of HeidelbergHeidelberg, Germany

**Keywords:** decision-making, option generation, creativity, long-term memory, affect, creative cognition, psychopathology

## Abstract

Which factors influence a human being’s ability to develop new perspectives and be creative? This ability is pivotal for any context in which new cognitions are required, such as innovative endeavors in science and art, or psychotherapeutic settings. In this article, we seek to bring together two research programs investigating the generation of creative options: On the one hand, research on option generation in the decision-making literature and, on the other hand, cognitive and clinical creativity research. Previous decision-making research has largely neglected the topic of generating creative options. Experiments typically provided participants with a clear set of options to choose from, but everyday life situations are less structured and allow countless ways to react. Before choosing an option, agents have to self-generate a set of options to choose from. Such *option generation* processes have only recently moved to the center of attention. The present study examines the creative quality of self-generated options in daily life situations. A student sample (*N* = 48) generated options for action in 70 briefly described everyday life scenarios. We rated the quality of the options on three dimensions of creativity- originality, feasibility, and divergence -and linked these qualities to option generation fluency (speed and number of generated options), situational features like the familiarity and the affective valence of the situation in which the options were generated, and trait measures of cognitive performance. We found that when situations were familiar to the participant, greater negative affective valence of the situation was associated with more originality and divergence of generated options. We also found that a higher option generation fluency was associated with a greater maximal originality of options. We complete our article with a joint research agenda for researchers in the decision-making field focusing on option generation and, on the other hand, researchers working on the cognitive and clinical aspects of creativity.

## Introduction

“In any given moment we have two options: To step forward into growth, or to step back into safety.”                    Maslow

Which factors influence a human being’s ability to develop new perspectives and be creative? This ability is pivotal for any context in which new cognitions are required (e.g., in psychotherapeutic settings) and something new needs to be developed (e.g., innovative endeavors in science and art). What is it that makes us revert to previously generated, existing options stored in our long-term memory (LTM), and what makes us step forward into thinking of new and creative options? Previous research on human decision-making has largely neglected the topic of creative option generation. Experiments have typically examined how people decide when facing an externally provided, multiple choice list of options (Glimcher and Rustichini, 2004; Rangel et al., 2008). This type of pre-structured decision situation sheds light on crucial decisional processes like option evaluation and option selection. However, generalizing these findings to decisions in everyday life poses a problem: Our complex world is full of decision situations that are not only far less structured but also under-constrained. They usually do not specify a finite and clear set of options to select from, but instead allow countless possible ways to react. Thus, in everyday life situations, before deciding what option to choose, one typically has to generate a set of options to choose from. Consider, for example, the following scenario: You are at home and want to cook when the power suddenly goes out. You can respond to the power blackout in endless ways, ranging from eating nothing at all to roasting marshmallows over a candle. The quality of the generated options determines the quality of the subsequent decision. Therefore, the preliminary process of *option generation* is of vital importance for decision-making in everyday life.

The present study aims at extending our research program on option generation in simple decision-making situations in everyday life (Kalis et al., 2008, 2013; Kaiser et al., 2013; Häusser et al., 2014). Several research groups have recently argued that the process of generating options should be added to existing decision-making models as a *pre-decisional phase* (e.g., Fellows, 2004; Kalis et al., 2008; Porcelli and Delgado, 2009; Kaiser et al., 2013). Lately, a growing number of publications has addressed the process of option generation on a theoretical level (e.g., Kalis et al., 2008; Smaldino and Richerson, 2012). An informative and concise review of publications on option generation is provided by Del Missier et al. (2015) who also present an overview of research on memory-based versus ideation-based views on the process of option generation. Memory-based views argue that option generation is based on the associative retrieval of choice options from LTM. By contrast, ideation-based views consider option generation as a process not only depending on associative retrieval but also requiring an ideation component. Recent research suggests that both retrieval and ideation processes play an important role for option generation, although it is far from clear how retrieval supports ideation and what kinds of ideation processes are used depending on the features of the situation in which the options are generated.

The present paper builds upon these theoretical approaches by also regarding situational features that dictate the generation and consideration of an option (Kalis et al., 2008): First, if the situation is *highly constrained*, one can choose only from the options defined by the situation (e.g., the abovementioned multiple choice task typically employed in decision-making research). Highly constrained situations can also be familiar situations. Second, in *unconstrained* situations that are *familiar*, one is most likely to generate options by retrieving them from LTM. Third, if the situation is *unconstrained* and *unfamiliar*, new options have to be generated which potentially involves creative processes. As Del Missier et al. (2015) also state: Besides basic associative memory retrieval, it is ideation processes (based on search and thinking processes) that contribute to option generation, mainly in this latter type of situation. In the present study, we take a closer look at these creative processes in the type of option generation occurring in unconstrained and unfamiliar situations as described above. In particular, we investigate which factors facilitate creative option generation processes by rating the quality of options among others with respect to their originality.

In order to investigate the process of option generation in simple, everyday life situations, we recently developed an experimental paradigm (Kaiser et al., 2013). Participants are presented with short vignettes of decision-making situations from various living domains and are required to name possible reactions (e.g., “You are in a new city and you have lost your way. What could you do?”). So far, this paradigm has been used to investigate option generation solely in terms of two fluency indicators, namely, how fast the generation process is initiated (generation onset time) and how many options are generated (quantity). Specifically, the quantity of generated options has been correlated with a cognitive test battery and, in a second part, with the results from functional magnetic resonance imaging (Kaiser et al., 2013). The present study extends this work by addressing the *quality* aspect of the generated options. To this end, options generated for an everyday scenario were rated in terms of their originality, feasibility, and divergence.

So far, the quality of self-generated options has been investigated by only a few studies. Previous work has defined quality in terms of the extent to which the option is useful for pursuing or achieving a clear goal. For instance, studies on option generation in sports conceived option quality as a situation-specific appropriateness when pursuing the goal of winning the competition (Johnson and Raab, 2003; Raab and Johnson, 2007; Hepler and Feltz, 2012). In a study by Ward et al. (2011), law enforcement officers naturally pursued the goal of ensuring the safety of the victim and of themselves. Similarly, a study by Del Missier et al. (2015) employed scenarios including clear goals (e.g., a domestic energy saving problem). Accordingly, the quality of the generated options was assessed by rating the options’ potential utility in effectively solving the problem. In stark contrast, everyday life situations are rarely characterized by a single and obvious goal, but rather by several and competing ones (Smaldino and Richerson, 2012), and the process of option generation is guided by the individuals’ idiosyncratic experiences and preferences. As an example, consider the following scenario of the option generation task by Kaiser et al. (2013): “Today is your first day of vacation on a tropical island. What could you do?” In this scenario, option generation is determined by the individual’s preferences rather than a clear goal that would be similar across individuals (e.g., whether the individual prefers to relax, do sports, or engage in cultural activities). In such real-world scenarios without a clear and unambiguous goal, it is impossible to rate the quality of the generated options in reference to their utility or to their effectiveness for solving a decision problem. Consequently, the present study defines an option’s quality in terms of its creative value.

Creativity is considered a multidimensional and heterogeneous concept (for an overview, see Runco, 2014). There is a general consensus on two defining features as suggested by Sternberg and Lubart (1999): A work is considered creative if it is *novel* (i.e., in the sense of *original* and unexpected) and *appropriate* (i.e., in the sense of usefulness). Whether something is appropriate or useful helps us to distinguish the creative from the absurd (Fink et al., 2014). Another aspect of creativity is the divergence of ideas, or the extent to which a set of generated options for a given situation differ from each other (Guilford, 1959). Furthermore, it has been argued that creativity arises from the interplay between different levels including, on the one hand, the person level which includes individual talents, traits and skills as well as their conative skills, and on the other hand, a social level in which the ideas and products presented are received and an evaluation or rating as to their creativity takes place (Schweizer, 2004, 2006). After decades of creativity research that struggled with conceptual heterogeneity, recent articles on the creativity topic reflect agreement on the fact that creativity needs to be broken down into various cognitive sub-processes (e.g., Akbari Chermahini and Hommel, 2011). In line with key aspects discussed in the creativity literature, we employ three quality dimensions: First, each option is rated in terms of how *original* it is, meaning the extent to which the option reflects an inventive idea in the situation, and, second, how *feasible* it is, that is, the extent to which the option is appropriate in the situation and, third, how *divergent* the set of options generated for this situation is, mirroring the extent to which the options generated for one situation differ from each other. The present work is based on the theoretical assumption that one of the decisive features of generating creative options is the ability to generate options that are new in the sense that they are different from what one would typically draw from LTM.

In the present study, we generated qualitative data complementing our quantitative data presented in Kaiser et al. (2013). This new qualitative data set represents an assessment of the creative quality of options; options were rated in terms of their divergence, originality, and feasibility. The present study was guided by three main questions: First, how quickly are options generated and how many options are generated when they are more creative compared to less creative ones? Second, under which situational circumstances do people generate more creative options? And third, what is the link between the creativity of the options generated and the cognitive performance of the option generator on various cognitive measures that could be related to option generation abilities?

Concerning option generation speed and creativity, it could be argued that people who generate more creatively also generate faster and more options due to, for example, higher general cognitive functioning acting as a mediator variable. On the other hand, it could also be argued that generating fewer options more slowly might lead to more creative options because it might take more time and cognitive resources to generate a creative option than a default option drawn from LTM. In line with these thoughts, two conflicting positions can be found in the option generation literature (Del Missier et al., 2015): Some researchers argue that generating fewer options leads to a higher average quality of the options (Johnson and Raab, 2003). Other researchers have argued that ‘quantity breeds quality’—that more options generated leads to a higher quality (Rietzschel et al., 2007). The findings of Del Missier et al. (2015) indicate that generating more options may increase the quality of the *best* option (in terms of its potential utility in solving a problem effectively), but decreases the mean quality of the options in the generated set. Evaluating the links between option generation fluency and option creativity is therefore an additional goal of the present study.

Concerning the situational circumstances of option generation, we consider two qualities of each situation potentially relevant to the fluency and creative quality of generated options. First, we consider the individual’s self-reported familiarity with the situation. Our previous study (Kaiser et al., 2013) revealed that significantly more options are generated in familiar situations compared to unfamiliar ones. Additionally, we showed that the options’ quantity across all scenarios was positively associated with LTM performance. Together, these findings suggest that retrieving options from memory is of relevance during option generation, and that this is especially the case in situations that are more familiar to the individual. It is therefore assumed that creative processes come into play most prominently when options cannot be retrieved from memory but have to be newly created. Hence, we expect a higher creativity of options generated in unfamiliar situations compared to familiar ones. Second, the present study examines the role of affective valence of the situation. Individuals reported the extent to which situations evoke positive or negative affect. This is of interest, since a broad range of studies empirically supported a link between affect and creative performance. Mood effects have been explained by motivational influences and the *feeling-as-information theory* by Schwarz (1990). Current affective states were considered as a cue for which processing strategy should be employed when performing creative tasks. It was argued that positive affect could be taken as a sign that everything is going well, and consequently lead to opting for a playful and effortless (i.e., more creative) approach to the task. Negative affect, in contrast, could be considered a warning signal, which evokes the motivation and willingness to apply a more effortful and detail-oriented approach that might be less creative. Indeed, a positive affective state has often been linked to more creativity (for meta-analyses, see Baas et al., 2008; Davis, 2009); however, in his meta-analysis, Davis (2009) also listed a series of studies that suggest the opposite, that is, that a negative affective state may facilitate creativity. Negative impulses have been argued to potentially function as a form of ‘negative social support’ to creative processes depending on the individual characteristics of the creator (Schweizer, 2007). For instance, individuals scoring high on the personality trait neuroticism may react completely different to negative versus positive forms of support to their creative endeavors than individuals scoring low on neuroticism. Also, complex problem solving researchers have shown that negative affect improves creative problem-solving in complex situations (Barth and Funke, 2010). In the present study, we focus on situational factors. Assuming that unpleasant scenarios in the option generation task tend to evoke negative affect while pleasant situations lead to positive affect, it will be interesting to determine under which situational conditions participants generate more creative options.

Our third research question addresses the link between the creative quality of the options generated and a set of cognitive functions measured by a cognitive test battery. The cognitive test battery provided measures of creative problem solving, creative idea generation, verbal fluency, cognitive set shifting, and LTM performance. In Kaiser et al. (2013), we found significant positive correlations between the number of generated options and performances in the LTM retrieval task and the category fluency task. Regarding the options’ quality, we now hypothesize the performance in creativity tests referring to divergent cognition capabilities to be positively associated with the generation of creative options. The cognitive set shifting test and verbal fluency test could reflect measures for the participants’ flexibility (i.e., the ability to switch mental sets easily; Brown, 1989) and fluency (i.e., the ability to generate grand numbers of ideas; Brown, 1989). Flexibility and fluency are generally considered to be associated with creative thinking (e.g., Torrance, 1990), which is why we hypothesize a positive association between performance in these two tests and the creativity of generated options. The correlation between the options’ creativity and LTM performance will be investigated in an exploratory manner.

## Materials and Methods

### Participants and Procedure

Our sample for this option generation quality study comprised 52 undergraduate students from the University of Göttingen from which we had to exclude four participants due to incomplete data (see also Kaiser et al., 2013 for our option generation quantity study). We carried out our analysis with the remaining 48 students (71% female) who were between the ages of 19 and 30 years (*M* = 22, *SD* = 2.37). The study was conducted in a single session during which participants first completed the option generation task followed by the cognitive test battery. The session lasted approximately 70 min. Prior to testing, the procedure was fully explained and all participants signed a written informed consent. For the time spent at the institute, participants either received extra course credit or a financial compensation of 10 €. The study received the institutional review board’s approval. With the sample size of *N* = 48, we are able to detect a significant two-tailed correlation rho = 0.4, given alpha = 0.05, with power (1–beta) = 0.80 (computed with G-Power 3 from Faul et al., 2007).

### Materials

#### Option Generation Task

**FIGURE 1:**
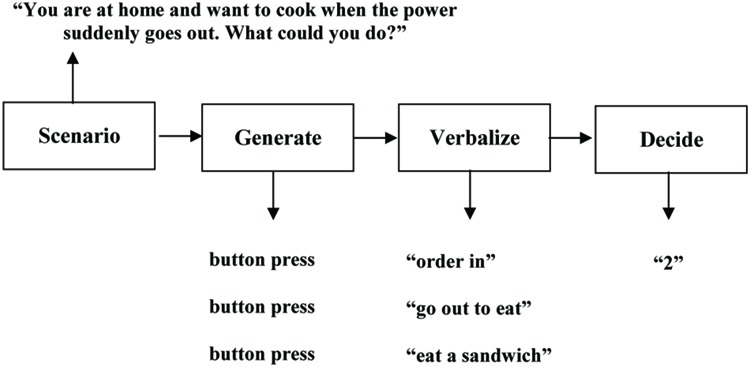
**Setup of an exemplary option generation trial with three generated options from which the second one was chosen**. Obtained and slightly modified from Kaiser et al. (2013).

Our option generation task (Kaiser et al., 2013) was implemented in Presentation (Neurobehavioral Systems Inc., Albany, NY, USA) and carried out on a computer with a microphone attached. The stimulus material consisted of 70 short scenarios reflecting ill-structured real-world situations (see Supplementary Material for a complete list of the situations). After being presented for 4 s on the notebook screen, the scenario disappeared and the word “generate” automatically appeared. Participants were instructed to immediately start with the mute generation of options regarding what one could do in this situation. Participants were given 8 s for this option generation phase, and were instructed to indicate the generation of each option by pressing a button on the notebook (mute option generation phase). In the following 8 s, participants had to verbalize the previously generated options (option verbalization phase). Next, the automatic change to the word “decide” asked participants to choose the preferred option out of the just generated ones (decision phase). The decision was made by pressing the number button on the notebook that reflected the preferred option’s generation rank (e.g., button “1” if the first generated option is chosen). An exemplary trial is displayed in **Figure 1** All trials were separated by a fixation cross. Participants were initially familiarized with the procedure by performing 10 test trials. After generating options in all 70 scenarios, participants were asked to rate the familiarity and the valence of each scenario on two bipolar 9-point Likert scales (*very unfamiliar* vs. *very familiar*; *very unpleasant* vs. *very pleasant*). Based on these ratings, we later carried out two median splits per participant, thus separating familiar from unfamiliar and pleasant from unpleasant scenarios.

*Generation onset time* was attained by measuring the time span between the appearance of the word “generate” on the notebook screen and the first pressing of the button, which indicated the first mutely generated option. The *number* of generated options was directly derived from the number of clicks in this mute option generation phase. In order to test whether the number of button presses in the mute option generation phase (i.e., the variable *number of generated options* used in the analyses) corresponds to the number of named options in the subsequent option verbalization phase, these numbers were correlated per scenario. The mean correlation across all 70 scenarios reached *r* = 0.62, *SD* = 0.12. Following Cohen's (1988) conventions to interpret the magnitude of correlation coefficients, this correlation represents a strong correlation. Since the number of button presses in the mute generation phase consistently exceeded the number of verbalized options, this result points to the fact that 8 s for the option verbalization phase may not have been long enough to name all of the generated options. However, note that participants were given no incentive to create a high number of options and, hence, ‘honest button presses’ could be expected, which means that it is very likely that participants only pressed the button in the mute option generation phase if they also had an idea for an option.

##### Creative option quality and rating procedures

The *creative quality of generated options* is based on the named options in the option verbalization phase. It was assessed by trained raters and followed a highly standardized protocol. Firstly, the non-redundant and valid options per scenario were derived from the transcriptions of the audio files. An option was labeled valid when it comprised an action (and not only an emotion or cognition), when it was concrete (and not abstract, e.g., “I would do something.”), and when it was feasible in the broadest sense (e.g., it is just impossible to turn back the time when you forgot your best friend’s birthday and undo your mistake). Secondly, two raters independently examined these valid and non-redundant options in terms of their divergence, originality, and feasibility. The *divergence* of all options generated per scenario, that is, the extent to which the options differ from each other, was assessed with a single score. In contrast, every option generated was rated separately in terms of its *originality* (the degree to which the option displays an inventive idea in the situation) and *feasibility* (the extent to which the option is realizable in the situation). These scores were averaged in order to derive the mean originality and mean feasibility of all options per scenario. The quality dimensions were rated on 5-point Likert scales. Two-way mixed, average-measures, consistency intra-class-correlations reached 0.42 for divergence, 0.46 for originality, and 0.47 for feasibility, which, according to Cicchetti (1994), indicates fair interrater-reliability. The mean values across raters were used to test our hypotheses.

#### Cognitive Correlates

We employed a set of five cognitive tests that provided measures for creative problem solving, creative idea generation, LTM performance, verbal fluency, and cognitive set shifting (Kaiser et al., 2013). The tests were either paper–pencil based or performed orally in interaction with a trained member of the research team. In particular, the following instruments were carried out.

##### Creativity Test: Remote Associating

A modified version of the *Remote Associates Test* (RAT; Mednick, 1962) was employed to assess creative problem solving. Each of the 16 trials comprised three stimulus words that were loosely associated to one another (e.g., “salt, deep, foam”). Participants’ task was to name a fourth word that linked the three (e.g., “sea”). The *number of correct answers* built the outcome measure. To complete the test, participants were given 15 min, which they could allocate freely across trials.

##### Creativity Test: Idea Generation

In order to measure creative idea generation, participants completed the *Product Names Task* (Marsh et al., 1999). This task required imagining oneself in an interview for a marketing job during which one had to create new labels for new products. Specifically, participants were asked to generate up to three new labels for each of the three product categories (pasta, nuclear element, and pain reliever). The instructions provided benchmark examples for each category (e.g., “Aspirin, Dolormin, Fortral, Ibuhexal, Tomaperin, Tramal”), along with the explicit request not to use or copy properties of the examples when forming new labels. Rubin et al. (1991) suggested that benchmark examples compromise the generation of novel and unique ideas. Thus, the measure for creative idea generation was derived from the *number of product labels* that did not have the same word ending as the examples (e.g., “-in,” “-al”). Participants were bound to no time limit when completing this task.

##### Verbal fluency and verbal set shifting

Four tasks of the *Regensburg word fluency test* (RWT; Aschenbrenner et al., 2000) were employed to capture participants’ verbal fluency: two letter fluency tasks and two category fluency tasks. The letter fluency tasks asked participants (1) to list as many words as possible that started with the letter “S” (measure for the variable *letter fluency with one letter*) and (2) to list as many words as possible that alternately started with the letters “G” and “R” (measure for the variable *letter fluency with two letters*). The variable *cost of change in letter fluency* was calculated by subtracting the number of words starting alternately with G/R from the number of words starting with an S and constitutes a measure for verbal set shifting. The category fluency tasks asked participants (3) to name as many words as possible belonging to the category “animal” (measure for the variable *category fluency with one category*) and (4) to alternately name as many words belonging to the categories “sport” and “fruit” (measure for the variable *category fluency with two categories*). The variable *cost of change in category fluency*, also a measure for verbal set shifting, was calculated by subtracting the number of alternately listed sports and fruits from the number of listed animals. Participants were given 60 s for completing each of the four tasks. A member of the research group counted the number of correct answers per task.

##### Numerical set shifting

In order to assess numerical set shifting, participants completed an adapted version of the *plus-minus task* developed by Spector and Biederman (1976). A single piece of paper showed three lists of 30 two-digit numbers. Instructions required participants to add 3 to each number on the first list, to subtract 3 from each number on the second list, and to alternately add and subtract 3 to and from the numbers on the third list. All answers were written down immediately and next to the corresponding number. A member of the research team recorded the time needed to complete each list. The *cost of numerical set shifting*, that is, the difference between the average time span for the addition and subtraction lists and the time span for the alternating list, was the variable of interest.

##### Long-Term memory performance

The *Verbal Learning and Memory Test* (VLMT; Helmstaedter et al., 2001) provided measures for LTM performance. Participants were asked to learn a list of 15 words that was read to them three times by a member of the research team. Kaiser et al. (2013) reduced the original presentation frequency of five times to three times in order to avoid ceiling effects in the highly educated and healthy sample. Following the three learning trials, participants were presented with an interference list. Free recall of the learned list was assessed directly after interference (measure for the variable *LTM performance after interference*) and a 15 min delay (measure for the variable *LTM performance after delay*). Two additional variables of interest were the *LTM loss through interference* (number of recalled words after learning phase minus number of recalled words after interference list) and *LTM loss through delay* (number of recalled words after learning phase minus number of recalled words after delay).

### Analytic Plan

Primary analyses were conducted in SAS PROC MIXED in two-level multilevel regression models (with 70 situations/items nested within each of the 48 individuals). First, we examined null multilevel models for each repeated measures variable (familiarity, valence, divergence, originality, maximum originality, feasibility, number of options, and generation onset time) and report *null model intercepts* and associated standard errors (which are a more accurate approximation of sample mean and standard deviation in the multilevel framework; Singer and Willett, 2003) and *intraclass correlations* (ICCs; i.e., the percentage of variance in the outcome attributable to between-person clustering; trait-like variance). We also examined Pearson correlations between aggregated person means for all variables. Next, we used multilevel models to examine the impact of familiarity, valence, and their interaction on the creative qualities of options (divergence, originality, and feasibility) and option generation fluency (number of options generated, generation onset time) for each situation *i*. In order to isolate the within-person variance in these situational predictors, each situation’s ratings of familiarity and valence were ipsatized within each person (also known as *centering within cluster*; Enders and Tofighi, 2007; e.g., the value of familiarity for situation *i* minus the *person’s mean* rating for familiarity across all of the 70 situations). This allows us to interpret effects of familiarity and valence in a personalized way for each individual, as these scores are centered around each person’s mean: values above 0 indicate that a given situation was rated by this individual as relatively more familiar (or pleasant) than other situations, whereas values below 0 indicate that a given situation was rated by this individual as relatively less familiar (or pleasant) than other situations. Finally, we also explore correlations among cognitive task results and person mean values for option quality and option generation fluency variables. Throughout, we employed a significance level of *p* = 0.01.

## Results

### Preliminary Analyses

Forty-eight individuals each contributed 70 situations to these analyses. **Table 1** describes null model intercepts (in lieu of sample means given our nested data structure; Singer and Willett, 2003) and ICCs. ICCs generally indicated that person-level clustering of familiarity, valence, divergence, originality, and feasibility were low, with just 1.5–8.4% of the variance in these ratings attributable to between-person factors. This indicates that these qualities of options tended to be more state-like than trait-like, and were mostly determined by situation-level factors. In contrast, option generation fluency variables were characterized by a larger degree of trait-like variance, with roughly 44% of the variance in number of options due to between-person factors, and roughly 32% of the variance in generation onset time due to between-person factors. Although the amount of between-person variance was small for some variables (see above), **Table 1** also describes the correlations among person means (each individual’s mean scores for each variable across all trials) for primary study variables. An individual’s mean divergence of options was positively associated with their mean originality of options (*r* = 0.51, *p* < 0.001), as well as mean number of options (*r* = 0.62, *p* < 0.001) and generation onset time (*r* = -0.64, *p* < 0.001). Mean feasibility was negatively correlated with mean divergence (*r* = -0.35, *p* < 0.01), mean originality (*r* = -0.42, *p* < 0.001), and the mean number of options generated (*r* = -0.40, *p* < 0.01). Mean number of options was negatively associated with mean generation onset time (*r* = 0.64, *p* < 0.001). In addition, average maximum originality of options was positively associated with mean divergence of options(*r* = 0.60, *p* < 0.001), mean number of options (*r* = 0.53, *p* < 0.001), and negatively with mean generation onset time (*r* = -0.46, *p* < 0.01).

**Table 1:** Between-person correlations among person means, null model intercepts, and intraclass correlations for option quality (divergence, originality, and feasibility) and option generation fluency (number of generated options and generation onset time in seconds).

Variable	*1*	*2*	*3*	*4*	*5*	*6*	*7*	NMI (*SE*)	ICC
1. Self-rated **Familiarity**								5.29 (0.12)	0.069
2. Self-rated **Valence**	-0.01							4.19 (0.10)	0.034
3. **Divergence** of options	0.23	-0.14						2.81 (0.04)	0.062
4. **Originality** of options	0.19	-0.14	0.51^∗∗^					1.61 (0.03)	0.084
5. Maximum originality of options	-0.10	-0.14	**0.60^∗∗^**	**0.53^∗∗^**				-	-
6. **Feasibility** of options	-0.03	-0.13	**-0.35^∗^**	**-0.42^∗∗^**	-0.30			3.97 (0.01)	0.015
7. Number of options	-0.02	0.12	**0.62^∗∗^**	0.11	**0.53^∗^**	**-0.40^∗^**		2.94 (0.11)	0.44
8. Generation onset time	0.07	0.16	**-0.64^∗∗^**	-0.13	**-0.46^∗^**	0.17	**-0.64^∗∗^**	1.74 (0.89)	0.32

*^∗^p < 0.01, ^∗∗^p < 0.001. NMI, null model intercept (in lieu of sample mean); ICC, intraclass correlation.Bolded values in the table indicate statistical significance at p < 0.01.*

### Creative Option Quality as a Function of Situational Features

Next, we utilized two-level regression models to investigate whether situational circumstances (familiarity, valence, and their interaction) influenced the creative qualities of generated options. Results of all models are shown in **Table 2** Random intercepts were included; random slopes were omitted due to lack of significance, which indicates that the fixed effects of familiarity and valence presented below did not vary strongly across individuals. However, inclusion of random slopes did not substantively alter any result presented below.

**Table 2:** Multilevel models: creative quality and option generation fluency in a given situation as a function of the participants’ PERSON-centered (CWP) familiarity and valence ratings for that situation.

	Situation-level outcome									
Situation-level predictors	Divergence		Originality		Feasibility		Number of options		Generation onset time	
	γ	*SE*	Γ	*SE*	γ	*SE*	γ	*SE*	γ	*SE*
Intercept	2.81	0.045	1.61	0.031	3.97	0.015	2.94	0.11	1.74	0.089
Familiarity_CWP_	0.032	0.020	-0.041^∗∗^	0.012	**0.045^∗∗^**	0.010	**0.058^∗∗^**	0.014	-0.006	0.015
Valence_CWP_	-0.050	0.020	-**0.028^∗∗^**	0.011	**0.19^∗∗^**	0.011	**0.026^∗∗^**	0.005	-0.007	0.015
Familiarity_CWP_ × Valence_CWP_	**-0.032^∗∗^**	0.009	-**0.013^∗∗^**	0.002	-**0.025^∗∗^**	0.006	0.006	0.013	0.024	0.011

*Bolded values in the table indicate statistical significance at p < 0.01.*

*^∗∗^p < 0.001.*

**FIGURE 2:**
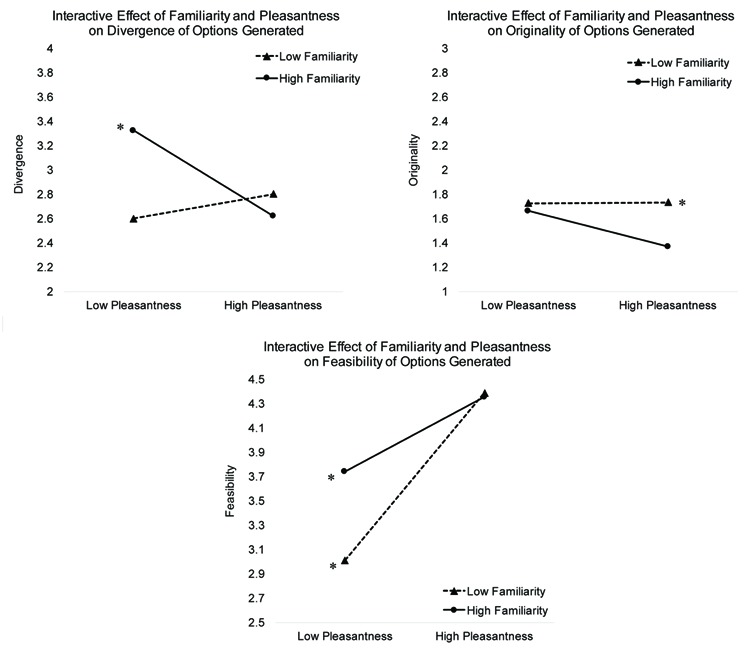
**Depiction of the interactive effects of familiarity and valence on creative qualities of generated options**. Asterisk denotes that the depicted simple slope is significant at ^∗^*p* < 0.01.+.

For all creative quality outcomes, a significant two-way interaction emerged between familiarity and valence (depicted in **Figure 2**). In order to understand the nature of these interactions we conducted follow-up simple slope analyses to determine the effects of situation valence on option qualities at high and low values of situation familiarity. Simple slopes that were significant are indicated by an asterisk in **Figure 2** Simple slope analyses for divergence and valence revealed similar patterns. At one standard deviation above the mean of person-centered familiarity, person-centered valence was associated with decreased divergence of options (Estimate for the effect of person-centered valence at +1 *SD above* the mean of person-centered familiarity: γ = -0.065, *SE* = 0.029, *t*(3208) = -2.26, *p* = 0.024) and decreased originality of options (Estimate for the effect of person-centered valence at +1 *SD above* the mean of person-centered familiarity: γ = -0.037, *SE* = 0.010, *t*(3208) = -2.21, *p* = 0.027). Effects of valence on divergence and originality were not significant at one standard deviation below the mean of familiarity (all *p*’s > 0.26). Simple slope analyses for feasibility indicated that person-centered valence predicted the feasibility of options at both high and low familiarity, although the impact of valence on feasibility was stronger at low familiarity (Estimate for the effect of person-centered valence at -1 *SD below* the mean of person-centered familiarity: γ = 0.22, *SE* = 0.012, *t*(3216) = 17.28, *p* < 0.0001; Estimate for the effect of person-centered valence at +1 *SD above* the mean of person-centered familiarity: γ = 0.17, *SE* = 0.015, *t*(3227) = 11.52, *p* < 0.0001).

### Option Generation Fluency as a Function of Situational Features

Next, we examined similar two-level regression models to investigate whether situational circumstances (familiarity, valence, and their interaction) influenced the option generation fluency. Results of models are also shown in **Table 2** There were no significant effects of familiarity, valence, or their interaction on generation onset time. However, there were main effects of both familiarity and valence on the number of options generated, with greater familiarity and valence each uniquely predicting a greater number of options generated.

### Correlations between Creative Option Quality, Option Generation Fluency, and Other Cognitive Measures

In order to examine the cognitive processes associated with the generation of creative options, we looked at the intercorrelations between measures in the cognitive test battery and each individual’s mean values for creative qualities (divergence, originality, and feasibility), and each individual’s mean values for option generation fluency (generation onset time, number of options generated). None of the tests were associated with divergence or feasibility. However, creative idea generation (as assessed by the *number of labels* named in the product names task) was associated with greater mean originality of options [*r*(48) = 0.39, *p* = 0.008] and poorer verbal set shifting (as assessed by the variable *cost of change in letter fluency*) was associated with less originality [*r*(48) = -0.33, *p* = 0.002]. Average maximum originality of options was not significantly associated with any of the cognitive tasks (*p* > 0.05)

Several of the cognitive tasks were also associated with option generation fluency. Average number of options generated per situation was positively associated with *letter fluency with two letters* [*r*(48) = 0.29, *p* = 0.007], *category fluency with one category* [*r*(48) = 0.34, *p* = 0.009], and *category fluency with two categories* [*r*(48) = 0.32, *p* = 0.006], and *LTM performance after interference* [*r*(48) = 0.28, *p* = 0.007], and negatively associated with *LTM loss through interference* [*r*(48) = -0.32, *p* = 0.004] and *LTM loss through delay* [*r*(48) = -0.29, *p* = 0.009]. Finally, *letter fluency with two letters* was associated with a faster generation onset time [*r*(48) = -0.32, *p* = 0.001]. **Table 3** presents an overview of the intercorrelations between performances in the cognitive test battery and option generation fluency, option quality, and reported familiarity and valence of the situation.

**Table 3:** Overview of the intercorrelations between generation fluency and creative quality of the options, familiarity and valence of the situation, and performances in the cognitive test battery.

	Mean self-rated familiarity	Mean self-rated pleasantness	Mean divergence of options	Mean originality of options	Maximum originality of options	Mean feasibility of options	Mean number of options	Mean generation onset time
RAT correct answers	0.00	-0.31	0.17	0.06	0.04	-0.03	-0.07	-0.23
Number of product labels	0.14	0.00	0.19	**0.39^∗^**	0.10	-0.12	-0.11	-0.11
Letter fluency with one letter	-0.07	0.02	0.13	-0.24	0.10	0.05	0.24	-0.27
Letter fluency with two letters	0.03	-0.19	0.26	0.00	0.10	-0.11	**0.29^∗^**	**-0.32^∗^**
Cost of change in letter fluency	-0.14	0.24	-0.12	**-0.33^∗^**	0.04	0.20	0.00	0.00
Category fluency with one category	-0.03	0.16	0.11	-0.02	0.32	-0.23	**0.34^∗^**	-0.19
Category fluency with two categories	-0.07	0.20	0.19	-0.09	0.23	-0.10	**0.32^∗^**	-0.19
Cost of change in category fluency	0.04	0.06	0.00	0.04	0.29	-0.21	0.19	-0.01
Cost of numerical set shifting	-0.02	-0.24	-0.27	-0.13	-0.10	0.09	-0.16	0.13
LTM performance after interference	-0.04	0.16	0.12	0.10	0.25	-0.10	**0.28^∗^**	-0.17
LTM performance after delay	-0.02	0.05	0.07	0.04	0.17	-0.05	**0.27**	-0.14
LTM loss through interference	0.00	-0.09	-0.25	-0.25	-0.31	0.20	**-0.32^∗^**	0.23
LTM loss through delay	-0.03	0.07	-0.18	-0.15	-0.17	0.13	**-0.29^∗^**	0.19

*Bolded values in the table indicate statistical significance at p < 0.01.^∗^p < 0.01.*

## Discussion

The present study aimed at extending our research program on option generation in simple decision-making situations in everyday life (Kalis et al., 2008, 2013; Kaiser et al., 2013; Häusser et al., 2014). We analyzed ratings of the *creative quality* (i.e., divergence, originality, and feasibility of options) of freely generated options in the context of our recently developed option generation paradigm (Kaiser et al., 2013).

Our first research question addressed the link between an individual’s mean creative quality of options and indicators of mean option generation fluency. High mean generation fluency (as indicated by a shorter mean generation onset time and a higher mean number of generated options) was positively associated with mean divergence of options and negatively associated with the mean feasibility of options. In other words, the faster and the more options individuals tend to generate, the more their options within a given scenario differ from each other, and the more difficult their typical generated option would be to enact. There were no significant between-person correlations between mean originality and mean option generation fluency, which, at first glance, would suggest that an individual’s quantitative performance in option generation does not strongly influence the creative quality of the options; importantly, however, a person’s average *maximum* originality was strongly positively associated with his or her average number of options generated and a shorter generation onset time. This finding is interesting since it informs the debate regarding the association between option generation fluency and option generation quality (Del Missier et al., 2015). Our results strengthen the position of those who argue that ‘quantity breeds quality’ (e.g., Osborn, 1953; Rietzschel et al., 2007) in that greater fluency was associated with greater maximum option originality. This is similar to the findings of Del Missier et al. (2015), who report that greater option generation fluency was linked to greater usefulness of the best option in a generated set.

Secondly, we investigated whether situational circumstances (i.e., situational familiarity, affective valence, and their interaction) influence the creative quality of the options generated. In general, less familiar situations (independently from their valence) were associated with more original and less feasible options. In situations with a more negative valence (independently from their familiarity), options were also more original and less feasible. In addition to these main effects of familiarity and valence, significant interactions between these two situation-level predictors emerged. In summary, situations with a negative valence (i.e., situations that evoke more negative affect for that individual) were associated with *more* creative options (in terms of both divergence and originality), *but only in situations that were relatively familiar to the individual*. In less familiar situations, valence of the situation did not significantly impact divergence or originality. Further, situations with a negative valence were associated with less feasible options, and this was particularly true when situations were not familiar to the individual. We also found a main effect concerning the quantity of the generated options and the valence of the situation. Familiarity and positive valence led to generating more options, but options were only more creative when the situation was marked by both familiarity and a negative valence. This pattern of findings is particularly interesting in the context of emotion and personality research, which found that inducing worry in people scoring high on neuroticism was supportive to creative processes (Leung et al., 2014). Also, in recent personality research on self-generated thought and neuroticism, it has been argued that people scoring high in the neuroticism dimension could be more creative (Perkins et al., 2015). Future work is needed to understand whether situations with a negative valence have a different impact on the creative performance of people scoring high or low on neuroticism.

Under challenging situational circumstances (as indicated by negative valence), the individual could have been more strongly motivated to engage in effortful cognitive processes, which could account for the finding of more original and less feasible options. At the same time, it seems that some degree of situational safety, indexed here by the familiarity of the situation, seems conducive to creative thought in negative affect-inducing situations. Future studies could further investigate the link between the options’ divergence and the situations’ valence, since several studies named by Davis (2009) suggest that a playful, effortless approach evoked by positive affect facilitates divergent thinking performance.

With regard to our third research question, we examined correlations between the creative quality of the options and a cognitive test battery with measures for remote associating, creative idea generation, verbal fluency and set shifting, numerical set shifting, and LTM performance. None of these tests were associated with the individual’s mean divergence and feasibility of generated options. In Kaiser et al. (2013), we reported that the performance in an LTM task was positively associated with the number of generated options. Our additional analyses revealed that performance in several verbal fluency and verbal set shifting tasks also shows a positive correlation with both indicators of option generation fluency.

Regarding the options’ originality, individuals with better verbal set shifting skills and individuals who show better creative idea generation listed more original options. While, we found the expected association between the options’ originality and the idea generation performance on the product names task (Marsh et al., 1999), we did not find an association with the remote association task, another creativity test used by creativity researchers. This is not surprising, because the latter creativity test captures a very different set of cognitive processes, which activates rather convergent than divergent cognitive processes. This once more confirms the relevance of one of the main caveats in creativity research: In the very heterogeneous creativity literature, one of the main problems is that creativity is operationalized in many different ways. Researchers need to be very clear about which specific cognitive processes supporting creativity they wish to address. Creative option generation clearly draws on divergent cognitive processes.

Finally, our findings make a contribution to the conceptual level of creativity research; we investigated the options’ creativity when creativity is defined as the *combination* of novelty and usefulness as proposed by Sternberg and Lubart (1999). The dimensions of divergence and originality represent novelty, while usefulness is represented by feasibility. Our analysis revealed that the definition of Sternberg and Lubart (1999) remains problematic: Options high in divergence and options high in originality turned out to be generally low in feasibility. This issue should be considered further as the definition of creativity continues to be clarified.

### Limitations

The results of the present study are challenged by a number of caveats. First, although it represents a substantial methodological improvement in several aspects, our option generation task (Kaiser et al., 2013) is not without limitations. The task marks an important step in the analysis of self-generated options in everyday life situations, but the question remains of how close to everyday life the employed situations actually are. The scenarios are only briefly described, and participants are provided with very little information in order to accurately represent option generation in under-constrained, real-life conditions. However, even though everyday life is characterized by few constraints, a great deal of information is available to us in daily life that may not be present in this laboratory task. Thus, the short descriptions of the scenarios might facilitate option generation since an effortful process of separating necessary from unnecessary information is not needed. On the other hand, one could argue that it becomes harder to generate options when information is missing. Consider the initial example: “You are at home and about to cook when the power suddenly goes out. What could you do?” When generating options, participants were missing information about what they were cooking, potential visitors they were expecting, the content of their refrigerator, and a host of other factors. Future studies could modify the task by enriching the scenarios with more details and subsequently compare the option generation processes in situations rich in detail with the ones in simplistic situations.

Furthermore, in the series of studies of which the present study forms a part, we had decided to choose a rather short generation time for each scenario (8 s) in order to follow the experimental paradigm of the fMRI study that was also part of this series (Kaiser et al., 2013, Study 2). This also meant that we were constrained to applying a research design including a mute generation phase followed by a verbalization phase in order to control for movement artifacts in the fMRI scanner for the related study. This time-restricted procedure may have limited the possibility of verbalizing all options previously generated in the mute generation phase as well as the generation of more creative options. This, in turn, may have benefitted memory retrieval processes rather than creative cognitive processes. Given the comparatively high number of scenarios, fatigue effects and decline in motivation may have also played a role. These factors should be kept in mind when looking at our results concerning the creative cognition variables. As we have argued in Section “Option Generation Task,” the number of button presses for options generated in the mute generation phase deviates somewhat from the number of named options in the option verbalization phase. The correlation we provided between the two numbers is only a proxy for their correspondence. Another limitation concerns the decision-phase: It may have been better to automatically record the participants’ verbal responses and display them on the screen in order to support participants in memorizing the order in which they verbalized their responses. By doing so, it would have been ensured that the button pressed to indicate the number of the preferred option reflects the real choice preferred for each trial. On the other hand, displaying the generated options on a screen might have decreased the ecological validity of our study, since in real life we rarely have to make decisions with all the choice options being displayed on a computer screen in front of us. A further potential limitation is that the sample size is not very large, and some weak but significant correlations may not have been detected due to relatively low power. As argued by Schönbrodt and Perugini (2013), although correlations converge to the population value with increasing sample size, the estimates tend to be inaccurate in small samples. The results of Monte-Carlo simulations by the researchers suggest that in typical studies the sample size should approach 250 for stable estimates (which, of course, is much larger than the sample size in our study). Finally, the product names task may not have been the ideal task for measuring the kind of divergent cognitive abilities benefiting option generation processes. Only performance measures like the alternative uses test have shown strong correlations with option generation fluency and other generation measures (see references in Del Missier et al., 2015). Future research on creative option generation processes would benefit from discussing in more depth the divergent cognition components underlying option generation processes in addition to basic cognitive and memory processes. The points raised above have been incorporated into our follow-up projects on the cognitive, affective and endocrinological aspects of creative option generation presented in the research agenda below.

## Conclusion and Research Agenda

Previous studies on decision-making have typically investigated how people choose from an externally provided clear set of options (i.e., multiple choice options). However, our complex world is filled with under-constrained decision-making situations that are unlikely to specify a finite and well-known set of options, instead allowing endless possible ways to react. Consequently, before being able to choose an option, we ourselves have to generate the set of options to choose from. The present study provides further insight into option generation processes in everyday life situations. Analyses revealed that generating more options does improve the quality of the best option in terms of its creativity and also that creativity changes as a function of situational features. These findings indicate that decision-making processes can be affected by situational influences in their initial option generation phase and that we may make more creative decisions if we make the effort to generate more options. Future studies should examine additional qualities of generated options, include additional situational features taking their affect-inducing qualities into account, and modify features of the option generation task in order to further approach the question of how options are generated. Furthermore, when it comes to the definition of creativity, the aspect of usefulness needs to be revisited.

In our follow-up studies to the work at hand we seek to deepen our insights into how situational influences affect the creative option generation process, particularly social affiliative aspects of the situation and the positive or negative affect they may induce. We also seek to better understand the role that individual level factors like a tendency toward negative affect may play in the creative process. This touches research on the link between creativity and psychopathology, which has been debated for thousands of years and has recently evolved into a scientific hotspot (Kaufman, 2014; Abraham, 2015). We propose that this work on creative option generation could also be relevant for clinical research in the realm of daily decision-making in line with recent research arguing that creativity can serve as a pathway out of maladaptive, psychopathological cognitions (Forgeard and Elstein, 2014). Additional studies will be needed in order to obtain a more complete understanding of the factors relevant in this link between creativity and psychopathological tendencies. In future studies, the interaction of person-level factors such as personality and situation-level factors should be further investigated. What personality type and which person-level profile benefit most from what type of situation when it comes to creative performance? Neuroticism, openness to experience and conscientiousness/obsessive-compulsive tendencies (Schweizer et al., 2006) as well as the need for cognition will be particularly interesting factors in this context.

Furthermore, we have recently started to include a completely new aspect of our neurocognitive research on creative processes: the role of neuroendocrinological factors. Which hormonal milieu is most conducive to higher cognition like creative option generation? In our study, we did not find a difference between men and women with respect to their propensity to generate creative options. However, there might be differences within the female menstrual cycle given that it has already been argued that estrogen could improve creative performance (Krug et al., 1994, 1996), a research avenue that unfortunately has not been pursued any further so far. Recently, the links between dopamine and divergent as well as convergent cognitive processes of creativity have been researched, where different constellations between phasic changes in dopamine and individually different tonic dopamine levels as well as mood have been suggested to be of key importance (Akbari Chermahini and Hommel, 2010, 2011, 2012). Only individuals with lower than median dopamine levels seemed to benefit from positive mood in the creative process (Akbari Chermahini and Hommel, 2012). Also, a u-shaped relationship between dopamine levels and creativity was found with an optimum mid-level of eye blink rates (EBRs, a clinical marker of dopamine levels) supporting flexibility in divergent thinking (Akbari Chermahini and Hommel, 2010). Furthermore, most recently, evidence has been presented that creativity can be predicted from interactions between genetic polymorphisms related to frontal (COMT) and striatal (DAT) dopaminergic pathways (Zabelina et al., 2016). In our follow-up studies to our study of creative cognition presented here, we bring the above research lines together looking at the role of the link between dopamine and estrogen as well as progesterone in the ups and downs of the menstrual cycle taking also the above-mentioned genetic factors into account and relating them to social factors like social support provided by significant others (e.g., partner, family) and creative cognitive performance. We would like to find out which are the best environments for creative work for different types of individuals with different hormonal profiles as they occur in different hormonal stages, such as different phases of the menstrual cycle. Also, could the more pronounced changes experienced by the minority of women with premenstrual affective disorders translate into greater fluctuation of creative cognition, just as more pronounced transitions between positive and negative affect have been argued to be conducive to creative cognition (Thys et al., 2014), as long as they are in a subclinical-mild to clinical-moderate range (Abraham, 2015)? How do creative states bordering on psychopathological phenomena of a transdiagnostic quality relate to psychoendocrinological states? We seek to shed light on other quality aspects of generated options like the social quality and relate them to the creative quality. We would like to find answers to questions like how much and what kind of social interaction is beneficial to creative processes and to whom depending on hormonally driven social cognitive styles, genetic as well as endocrinological vulnerabilities? While we did not find an overall difference in creative option generation performance between the sexes in the study presented here, we did find differences in option generation performance in women between menstrual cycle phases in another study (Schweizer et al., 2016). How? This is far from understood. We wish to invite our fellow scientists in the fields of cognitive and clinical research on creativity as well as option generation research in decision-making to join us in a collective effort of using our own creative cognition for finding answers to the above questions.

## Author Contributions

All authors listed, have made substantial, direct and intellectual contribution to the work, and approved it for publication.

## Conflict of Interest Statement

The authors declare that the research was conducted in the absence of any commercial or financial relationships that could be construed as a potential conflict of interest.
